# Dr. E. M. Hall

**Published:** 1846-06

**Authors:** 


*Obituary.*—We are called upon to perform the painful task of announcing the decease of Dr. E. M. Hall, who died at Goldsboro', N. C., on the 24th of May, 1846, in the thirty-first year of his age, of phthisis pulmonalis, after a short illness, in the triumphs of Christian faith. His amiable disposition and gentlemanly deportment had secured for him the friendship and esteem of all who knew him, and his untimely death is a source of deep sorrow to his relatives, and regret to a large circle of friends.

Dr. Hall graduated in the Medical Department of the University of Maryland, in the spring of 1834, and for several years was a successful practitioner of medicine; but his health becoming impaired, he retired from practice, and engaged in the study of dentistry, attended lectures in the Baltimore College of Dental Surgery, and graduated in the spring of 1845. He then engaged in the practice of this department of the curative art, and continued to exercise its duties until a short time previously to his death.

